# Associations of dairy and fiber intake with circulating odd-chain fatty acids in post-myocardial infarction patients

**DOI:** 10.1186/s12986-019-0407-y

**Published:** 2019-11-13

**Authors:** Kamalita Pertiwi, Leanne K. Küpers, Anne J. Wanders, Janette de Goede, Peter L. Zock, Johanna M. Geleijnse

**Affiliations:** 1 Division of Human Nutrition and Health, Wageningen University, PO Box 17, 6700 AA Wageningen, The Netherlands; 2 Future Health & Wellness, Unilever R&D, Vlaardingen, The Netherlands

**Keywords:** Plasma fatty acids, Pentadecanoic acid, Margaric acid, Biomarkers, Food frequency questionnaire

## Abstract

**Background:**

Circulating odd-chain fatty acids pentadecanoic (15:0) and heptadecanoic acid (17:0) are considered to reflect dairy intake. In cohort studies, higher circulating 15:0 and 17:0 were associated with lower type 2 diabetes risk. A recent randomized controlled trial in humans suggested that fiber intake also increased circulating 15:0 and 17:0, potentially resulting from fermentation by gut microbes. We examined the associations of dairy and fiber intake with circulating 15:0 and 17:0 in patients with a history of myocardial infarction (MI).

**Methods:**

We performed cross-sectional analyses in a subsample of 869 Dutch post-MI patients of the Alpha Omega Cohort who had data on dietary intake and circulating fatty acids. Dietary intakes (g/d) were assessed using a 203-item food frequency questionnaire. Circulating 15:0 and 17:0 (as % of total fatty acids) were measured in plasma phospholipids (PL) and cholesteryl esters (CE). Spearman correlations (*r*_*s*_) were computed between intakes of total dairy, dairy fat, fiber, and circulating 15:0 and 17:0.

**Results:**

Patients were on average 69 years old, 78% was male and 21% had diabetes. Total dairy intake comprised predominantly milk and yogurt (69%). Dairy fat was mainly derived from cheese (47%) and milk (15%), and fiber was mainly from grains (43%). Circulating 15:0 in PL was significantly correlated with total dairy and dairy fat intake (both *r*_*s*_ = 0.19, *p* < 0.001), but not with dietary fiber intake (*r*_*s*_ = 0.05, *p* = 0.11). Circulating 17:0 in PL was correlated both with dairy intake (*r*_*s*_ = 0.14 for total dairy and 0.11 for dairy fat, *p* < 0.001), and fiber intake (*r*_*s*_ = 0.19, *p* < 0.001). Results in CE were roughly similar, except for a weaker correlation of CE 17:0 with fiber (*r*_*s*_ = 0.11, *p* = 0.001). Circulating 15:0 was highest in those with high dairy intake irrespective of fiber intake, while circulating 17:0 was highest in those with high dairy and fiber intake.

**Conclusions:**

In our cohort of post-MI patients, circulating 15:0 was associated with dairy intake but not fiber intake, whereas circulating 17:0 was associated with both dairy and fiber intake. These data suggest that cardiometabolic health benefits previously attributed to 17:0 as a biomarker of dairy intake may partly be explained by fiber intake.

## Introduction

Circulating odd-chain fatty acids (OCFA) pentadecanoic (15:0) and heptadecanoic acid (17:0) have been used as biomarkers of dairy and dairy fat intake in observational studies [1, 2]. The reason is that fatty acids primarily coming from exogenous sources are usually considered good candidates as biomarkers for intake [3] and more objective than self-reported dietary assessment. Since OCFA are considered to be solely produced in rumen of ruminants and cannot be produced by the human body, they have been proposed as good candidate biomarkers for dairy fat and/or total dairy intake [4–6]. A recent meta-analysis of 18 observational studies showed that intake of dairy and dairy fat were more correlated with circulating proportions of 15:0 (with correlation coefficients (*r*) of 0.20 and 0.33, respectively) than 17:0 (*r* = 0.10 and 0.19, respectively) [1].

Higher circulating 15:0 and 17:0 have been associated with lower risk of cardiometabolic outcomes, such as type 2 diabetes [7–9] and cardiovascular disease [10–12]. A recent pooled analysis of 16 prospective cohort studies showed that higher circulating 15:0 and 17:0 were associated with 20 and 35% lower risk of type 2 diabetes, respectively, by comparing individuals with circulating 15:0 or 17:0 in the 90th to those in the 10th cohort-specific percentile [2]. In these studies, the beneficial associations between circulating 15:0 and 17:0 and cardiometabolic risk were often attributed to dairy intake only.

In a randomized controlled trial in 16 healthy participants, supplementation of inulin, a fermentable fiber, increased the level of circulating OCFA in plasma phospholipids (PL) [13]. In the European Prospective Investigation into Cancer and Nutrition (EPIC) Inter-Act study, both intake of dairy and fruits and vegetables, which are important sources of fiber, were associated with the sum of circulating 15:0 and 17:0 in PL, with correlations between 0.1–0.2 [7]. Another observational study showed associations of circulating PL 15:0 with ruminant meat intake (*r* = 0.4) and dairy fat (*r* = 0.5) [14]. These data suggest that circulating OCFA may not only reflect dairy and/or dairy fat intakes, but also intakes of other foods.

Our primary aim in the present analyses was to examine the associations of dairy, dairy fat and fiber intakes with circulating 15:0 and 17:0 in a cohort of Dutch post-myocardial infarction patients.

## Methods

### Study design and population

Cross-sectional analyses were carried out in baseline data of the Alpha Omega Cohort (years 2002–2006) as described previously [15, 16]. This cohort consists of 4837 Dutch patients aged 60 through 80 who had a myocardial infarction (MI) up to 10 years before study enrollment. The medical ethics committee at the Haga Hospital (The Hague, The Netherlands) approved the study and all patients provided written informed consent. The present analysis included 869 patients with complete and reliable data on dietary intake and plasma fatty acids both in PL and cholesteryl esters (CE) **(**Additional file 1: Figure S1).

### Dietary assessment

Habitual dietary intake was assessed with a 203-item food frequency questionnaire (FFQ) which was an extended version of a previously validated FFQ to estimate fatty acids and cholesterol intake [17, 18]. The FFQ contained 42 items on various dairy products for which questions were grouped by fat contents: whole, semi-skimmed or skimmed. Total dairy included intake of milk, yogurt, cheese, dairy desserts, cream, milk for coffee and creamers, butter and ice-cream. Intakes of different dairy products in grams/day were calculated by multiplying consumption frequencies and portion sizes. Intake of milk and creamers from non-dairy sources such as soy milk and non-dairy creamers were not included. Daily intake of total energy (kcal/d; including alcohol), foods (g/d) and nutrients, including dairy fat (g/d), were calculated after linkage of intake data to the 2006 Dutch food composition table (NEVO) [19].

Dairy products were divided in two groups based on their fat content. Low-fat dairy included semi-skimmed (fat content ≤1.8 g/100 ml) and skimmed (fat content ≤1.5 g/100 ml) milk, buttermilk, yogurt drink and other low-fat dairy products. High-fat dairy included full-fat dairy products (fat content > 3.0 g/100 g for solids or > 1.8 g/100 ml for liquids), all types of cheese, dairy-based coffee creamer, butter, cream and ice-cream **(**Additional file 1: Table S1).

Total fiber intake was calculated by summing up intakes of fiber from all dietary sources (excluding dietary supplements) (Additional file 1: Table S2). Dietary fiber included plant constituents that cannot be digested by human enzymes in stomach and small intestine, e.g. lignin, cellulose, hemicellulose and pectin, as measured using the recommended Association of Official Analytical Chemists method [19]. Total meat included beef, pork, chicken, turkey, lamb, mutton and other meats. Ruminant meat included intake of beef, lamb and mutton. Total fish included fatty fish, lean fish and shellfish. Intakes of foods and food groups were expressed as g/d.

### Data collection on risk factors

Information about demographic factors, lifestyle characteristics and medication use were obtained by self-administered questionnaires as previously described [15]. The validated Physical Activity Scale for the Elderly was used to assess the physical activity level of the patients [20]. Alcohol intake (ethanol, in g/d) was assessed with the FFQ and categorized as ‘no’ (0 g/d), ‘low’ (> 0 to 10 g/d), ‘moderate’ (> 10 to 20 g/d in women; > 10 to 30 g/d in men) or ‘high’ (> 20 g/d in women; > 30 g/d in men). Medications were coded according to the Anatomical Therapeutic Chemical Classification system, with codes C10AA and C10B for statins, C02 for antihypertensive drugs and A10 for antidiabetic drugs [21]. Physical examination took place at home or in the outpatient clinic by trained research nurses. Body mass index was calculated from measured weight divided by height squared (kg/m^2^). A fasted or non-fasted venous blood sample (30 mL) was drawn from each patient. Fasting was defined as at least 8 h since the last meal. Serum lipids and plasma glucose were measured at baseline using standard kits as previously described [15]. Diabetes was considered present if patients reported a physician’s diagnosis of diabetes, used anti-diabetes medication, or had plasma glucose ≥7.0 mmol/L (fasting) or ≥ 11.1 mmol/L (non-fasting) [22]. Blood samples were stored at − 80 °C for future analysis.

### Measurement of odd-chain fatty acids

Circulating OCFA 15:0 and 17:0 were measured in plasma PL and CE. Laboratory procedures for measuring fatty acid composition in plasma PL and CE in the Alpha Omega Cohort have been described in detail previously [23]. In short, plasma total lipids from 10 mL EDTA blood samples were extracted and separated into plasma PL and CE fraction of fatty acids by using solid phase extraction method. Subsequently, fatty acids in each fraction were trans-esterified into fatty acid methyl esters. Fatty acid composition in plasma PL and CE were measured using gas chromatography equipped with a flame ionization detector. In total, 38 fatty acids were detected by comparison to pure fatty acid standards. For each plasma fraction, circulating individual fatty acids were expressed as proportion of total fatty acids measured (% total fatty acids).

### Statistical analysis

Patient characteristics were expressed as crude means (± standard deviation, SD) or medians (interquartile range, IQR) for continuous variables and numbers (percentage) for categorical variables. Spearman’s rank correlation (*r*_*s*_) was used to measure the associations of total dairy, dairy fat and total fiber intakes with circulating 15:0 and 17:0, for PL and CE separately. Partial correlation coefficients were calculated with adjustment for age, sex and total energy intake and their 95% confidence intervals (CI) were obtained by Fisher’s z transformation. Additionally, we assessed the dose-response association of dairy or fiber intake with circulating 15:0 and 17:0 using restricted cubic splines with three knots placed at 5th, 50th and 95th percentile, adjusted for age, sex and total energy intake. To examine whether correlations between dairy and 15:0 and 17:0 were modified by dietary fiber, partial correlations were obtained in strata of low and high total fiber intake (< 21.2 g/d versus ≥21.2 g/d, based on median energy-adjusted intake). Likewise, partial correlations between dietary fiber and circulating OCFA were calculated for strata of energy-adjusted total dairy intake (median = 301 g/d) and dairy fat (median = 12.0 g/d). We also assessed circulating 15:0 and 17:0 in combined subgroups of dairy and fiber intake: ‘low dairy-low fiber’, ‘low dairy-high fiber’, ‘high dairy-low fiber’ and ‘high dairy-high fiber’ by using generalized linear models (normal distribution, identity link function) with robust standard errors, also adjusted by age, sex and total energy intake.

Because ruminant meat and fish can contain 15:0 and 17:0, we also calculated partial correlations of intakes of total meat, ruminant meat and total fish with circulating 15:0 and 17:0 in PL and CE. SAS 9.4 (Cary, NC, USA) was used for all analyses, and a two-sided *p*-value < 0.05 was considered as statistically significant. The %RCS_Reg macro was used to perform restricted cubic splines analyses [24].

## Results

Patients were on average 69.2 (SD = 5.7) years old and enrolled in the study ~ 3 year after MI (Table 1). Of the cohort, 78% were male, 21% had diabetes and 24% was obese. Most patients (> 85%) used cardiovascular medication, including statins and anti-hypertensive drugs.

**Table 1:** Characteristics of patients in the subsample of Alpha Omega Cohort for the present analysis^a^

	Value
Age (y)	69.2 ± 5.7
Men, *n* (%)	678 (78.0)
Body mass index (kg/m^2^)	27.8 ± 4.0
Obese, *n* (%)^b^	205 (23.6)
Smoking status, *n* (%)^c^	
Never	150 (17.3)
Former	595 (68.6)
Current	123 (14.2)
Physical activity, *n* (%)^c,d^	
Low	327 (37.9)
Medium	356 (41.3)
High	179 (20.8)
Alcohol intake, *n* (%)^e^	
No	58 (6.7)
Low	464 (53.4)
Moderate	223 (25.7)
High	124 (14.3)
Time since last myocardial infarction (y)	3.0 (1.4–5.9)
Prevalent diabetes, *n*(%)^f^	186 (21.4)
Medication use, *n* (%)	
Statins	772 (88.8)
Anti-hypertensive	818 (94.1)
Anti-diabetes	129 (14.8)
Serum lipids (mmol/L)	
Total cholesterol^c^	4.43 ± 0.89
LDL cholesterol^g^	2.25 ± 0.75
HDL cholesterol^c^	1.37 ± 0.35
Triglycerides^c^	1.59 (1.18–2.31)
Odd-chain fatty acids (% total fatty acids)	
Phospholipids 15:0	0.14 (0.12–0.16)
Phospholipids 17:0	0.38 ± 0.08
Cholesteryl esters 15:0	0.16 ± 0.04
Cholesteryl esters 17:0	0.08 (0.07–0.10)

*HDL* high-density lipoprotein, *LDL* low-density lipoprotein, *MET* metabolic equivalent

^a^Values are mean ± standard deviation or median (interquartile range) or *n* (%)

^b^Obesity defined as body mass index ≥30 kg/m^2^

^c^Missing values for < 1% of patients

^d^Categorized as ‘low’: no activity or only light activity (≤ 3 METs), ‘medium’: > 0 to < 5 days per week of moderate or vigorous activity (> 3 METs), and ‘high’: ≥5 days per week of moderate or vigorous activity

^e^Categorized as ‘no’: 0 g/d, ‘low’: > 0 to 10 g/d, ‘moderate’: > 10 to 20 g/d for women and > 10 to 30 g/d for men, and ‘high’: > 20 g/d for women and > 30 g/d for men

^f^Defined as a self-reported physician’s diagnosis, use of anti-diabetes medication, or plasma glucose ≥7.0 mmol/L (fasting) or ≥ 11.1 mmol/L (non-fasting)

^g^Missing values for 44 patients for LDL cholesterol

Median total dairy intake was 294 g/d, which was predominantly low-fat dairy (73%) (Table 2). Total dairy mainly comprised milk (median = 150 g/d), followed by yogurt (56 g/d), dairy desserts (e.g. quark and custard; 22 g/d), and cheese (20 g/d). Median intake of dairy fat was 11.1 g/d (19% of the total fat intake), of which 1.3 g/d was from milk and 4.5 g/d was from cheese. Median total fiber intake was 21.0 g/d, which was mainly derived from grains (43% of total fiber). Median intakes of total meat, ruminant meat and total fish were 59 g/d, 17 g/d and 14 g/d, respectively.

**Table 2:** Reported intake of dairy products, dairy fat, total fiber and other foods in AOC subsample^a, b^

	Mean ± SD	Median (Q1-Q3)
Total dairy	343 ± 248	294 (191–418)
High-fat dairy	92 ± 98	65 (37–113)
Low fat dairy	251 ± 224	201 (110–321)
Milk	161 ± 183	150 (21–167)
Yogurt	75 ± 85	56 (18–128)
Cheese	26 ± 26	20 (9–32)
Cream	1 ± 2	0 (0–1)
Butter	1 ± 5	0 (0–0)
Dairy desserts	45 ± 66	22 (13–58)
Ice-cream	10 ± 19	9 (0–13)
Milk for coffee and creamers	23 ± 52	5 (0–25)
Dairy fat	13.6 ± 9.8	11.1 (7.3–17.2)
Fat from milk	2.1 ± 3.0	1.3 (0.3–2.5)
Fat from yogurt	0.8 ± 1.2	0.3 (0.1–1.4)
Fat from cheese	6.4 ± 6.4	4.5 (2.3–7.7)
Fat from cream	0.2 ± 0.4	0.0 (0.0–0.3)
Fat from butter	0.9 ± 4.0	0.0 (0.0–0.0)
Fat from dairy desserts	1.6 ± 2.6	0.5 (0.0–2.0)
Fat from ice-cream	0.7 ± 1.4	0.7 (0.0–1.0)
Fat from milk for coffee and creamer	0.9 ± 1.5	0.3 (0.0–1.2)
Total fiber	21.5 ± 6.9	21.0 (16.7–25.1)
Total meat	52 ± 34	59 (30–72)
Ruminant meat	18 ± 15	17 (6–26)
Total fish	17 ± 19	14 (4–17)

*AOC* Alpha Omega Cohort, *Q* quartile

^a^Intake (not energy-adjusted) is reported in grams/day

^b^Individual dairy products were listed in Additional file 1: Table S1

Circulating proportions of 15:0 were comparable in PL and CE, while for 17:0, proportions in PL were higher than in CE (Table 1). Proportions of 15:0 and 17:0 within each fraction were correlated (crude *r*_*s*_ in PL = 0.56, in CE = 0.47; all *p* < 0.001; data not in Table).

### Associations of circulating 15:0 and 17:0 with dietary intake

Circulating 15:0 in both PL and CE was significantly correlated with intake of total dairy and dairy fat; partial *r*_*s*_ ranged from 0.19 to 0.26 (Table 3). Circulating 15:0 in both PL and CE were not significantly associated with total fiber intake (partial *r*_*s*_ ≤ 0.05). Circulating 17:0 in PL was significantly correlated with intakes of total dairy (partial *r*_*s*_ = 0.14), dairy fat (partial *r*_*s*_ = 0.11), and total fiber intake (partial *r*_*s*_ = 0.19). Correlations with 17:0 in CE were similar for total dairy, dairy fat and total fiber. However, the correlation of total fiber with 17:0 in CE (partial *r*_*s*_ = 0.11) was smaller than for PL. Dose-response analysis using restricted cubic splines showed that associations of PL 15:0 and 17:0 with total dairy were non-linearly shaped. For CE, only the association between 15:0 and total dairy intake was non-linear (Additional file 1: Figure S2–S4). No correlations were observed between circulating OCFA and other food groups, except for a weak correlation of 15:0 in CE with total fish intake (partial *r*_*s*_ = 0.09) (Table 3), which persisted when only the fish eaters (*n* = 680) were included in the analysis (partial *r*_*s*_ = 0.10; not shown in Table). Additional adjustments for medications and presence of type 2 diabetes did not appreciably change the results **(**Additional file 1: Table S3). However, in sensitivity analysis including only patients without type 2 diabetes (*n* = 683), PL 17:0 was weakly correlated with ruminant meat intake (*p* = 0.013) and the correlation of CE 15:0 with fish intake was no longer statistically significant **(**Additional file 1: Table S4).

**Table 3:** Correlations between intakes of dairy, fiber and other foods and circulating 15:0 and 17:0^a^

	Fractions	Pentadecanoic acid (15:0)		Heptadecanoic acid (17:0)	
		Crude	Partial	Crude	Partial
Dairy					
Total dairy	Phospholipids	0.19 (0.12, 0.25)^***^	0.19 (0.12, 0.25)^***^	0.14 (0.08, 0.21)^***^	0.14 (0.08, 0.21)^***^
	Cholesteryl esters	0.22 (0.15, 0.28)^***^	0.23 (0.17, 0.30)^***^	0.11 (0.04, 0.17)^**^	0.14 (0.07, 0.20)^***^
Dairy fat	Phospholipids	0.17 (0.10, 0.23)^***^	0.19 (0.13, 0.26)^***^	0.10 (0.03, 0.16)^**^	0.11 (0.05, 0.18)^***^
	Cholesteryl esters	0.22 (0.16, 0.29)^***^	0.26 (0.20, 0.32)^***^	0.10 (0.03, 0.16)^**^	0.12 (0.05, 0.18)^***^
Total fiber	Phospholipids	0.01 (− 0.06, 0.08)	0.05 (− 0.01, 0.12)	0.13 (0.07, 0.20)^***^	0.19 (0.12, 0.25)^***^
	Cholesteryl esters	0.00–0.07, 0.07)	0.03 (−0.04, 0.10)	0.09 (0.02, 0.15)^**^	0.11 (0.04, 0.18)^**^
Other foods					
Total meat	Phospholipids	−0.02 (− 0.09, 0.04)	−0.01 (− 0.08, 0.05)	0.01 (− 0.06, 0.07)	0.02 (− 0.05, 0.08)
	Cholesteryl esters	−0.05 (− 0.11, 0.02)	−0.04 (− 0.10, 0.03)	0.01 (− 0.05, 0.08)	0.02 (− 0.05, 0.08)
Ruminant meat	Phospholipids	0.06 (− 0.01, 0.12)	0.06 (− 0.01, 0.13)	0.06 (− 0.01, 0.12)	0.06 (− 0.00, 0.13)
	Cholesteryl esters	0.05 (− 0.01, 0.12)	0.06 (− 0.01, 0.12)	0.04 (− 0.03, 0.10)	0.04 (− 0.03, 0.11)
Total fish	Phospholipids	−0.00 (− 0.07, 0.06)	0.01 (− 0.05, 0.08)	0.06 (− 0.01, 0.12)	0.06 (− 0.01, 0.13)
	Cholesteryl esters	0.07 (0.00, 0.13)^*^	0.09 (0.02, 0.15)^*^	0.02 (− 0.04, 0.09)	0.02 (− 0.05, 0.09)

^*^p < 0.05; ^**^p < 0.01; ^***^p < 0.001

^a^Crude: Spearman’s correlation of intakes (g/d) with circulating 15:0 or 17:0 in phospholipids or cholesteryl esters (% of total fatty acids); partial: Spearman’s correlation of energy-adjusted intakes (g/d) with circulating 15:0 or 17:0 in phospholipids or cholesteryl esters (% of total fatty acids), adjusted for age, sex, total energy intake

**Fig. 1:**
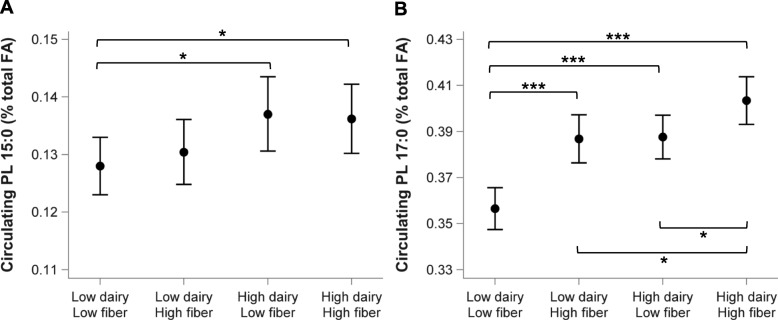
Circulating phospholipids 15:0 (**a**) and 17:0 (**b**) in dairy and fiber intake strata. **a** Low dairy intake: < 301 g/d; high dairy intake: ≥301 g/d, **b** Low fiber intake: < 21.2 g/d; high fiber intake: ≥21.2 g/d, ^*^p < 0.05; ^**^p < 0.01; ^***^p < 0.001.

In patients with a high fiber intake, partial correlations of dairy intake with circulating OCFA were smaller than in those with a low fiber intake (Table 4). In patients with a high total dairy intake, partial correlations of total fiber intake with circulating 17:0 in PL and CE were smaller than in patients with a low total dairy intake, and total fiber intake was not correlated with 15:0 in patients with higher intakes of total dairy or dairy fat (Table 5). Assessment of the circulating 15:0 and 17:0 in different strata of dairy and fiber showed that patients with low or high fiber intake had similar mean circulating PL 15:0 when their dairy intake was high (Fig. 1). The highest mean circulating PL 17:0 was observed for patients with both high dairy and high fiber intake, while patients with either low dairy-high fiber intake or high dairy-low fiber intake had similar mean circulating PL 17:0. Results in CE were similar (data not shown).

**Table 4:** Partial correlations between dairy intake and circulating 15:0 and 17:0 in strata of fiber intake^a, b^

	Pentadecanoic acid (15:0)		Heptadecanoic acid (17:0)	
	Phospholipids	Cholesteryl esters	Phospholipids	Cholesteryl esters
Total dairy				
Low fiber (*n* = 438)	0.22 (0.13, 0.30)^***^	0.27 (0.18, 0.36)^***^	0.20 (0.11, 0.29)^***^	0.16 (0.07, 0.25)^***^
High fiber (*n* = 431)	0.17 (0.07, 0.26)^***^	0.19 (0.10, 0.28)^***^	0.10 (0.00, 0.19)^*^	0.11 (0.02, 0.20)^*^
Dairy fat				
Low fiber (*n* = 438)	0.23 (0.14, 0.31)^***^	0.32 (0.23, 0.40)^***^	0.20 (0.10, 0.28)^***^	0.18 (0.08, 0.27)^***^
High fiber (*n* = 431)	0.18 (0.09, 0.28)^***^	0.22 (0.12, 0.30)^***^	0.09 (−0.00, 0.19)	0.09 (− 0.01, 0.18)

^*^p < 0.05; ^***^p < 0.001

^a^Spearman’s correlation of energy-adjusted dietary intakes (g/d) with circulating 15:0 or 17:0 (% of total fatty acids), partially adjusted for age, sex, total energy intake

^b^“Low fiber” referring to subsample of patients with energy-adjusted total fiber intake below median intake of total sample and “high fiber” to patients with energy-adjusted total fiber intake at median intake or higher. Median energy-adjusted total fiber intake = 21.2 g/d

**Table 5:** Partial correlations between fiber intake and circulating 15:0 and 17:0 in strata of dairy intake^a, b, c^

Dietary intake	Pentadecanoic acid (15:0)		Heptadecanoic acid (17:0)	
	Phospholipids	Cholesteryl esters	Phospholipids	Cholesteryl esters
Total fiber				
Low total dairy (*n* = 434)	0.10 (0.01, 0.19)^*^	0.10 (0.01, 0.20)^*^	0.25 (0.16, 0.34)^***^	0.13 (0.03, 0.22)^**^
High total dairy (*n* = 435)	0.02 (− 0.08, 0.11)	−0.01 (− 0.11, 0.08)	0.13 (0.04, 0.22)^**^	0.10 (0.00, 0.19)^*^
Total fiber				
Low dairy fat (*n* = 434)	0.15 (0.06, 0.24)^**^	0.17 (0.08, 0.26)^***^	0.26 (0.17, 0.35)^***^	0.18 (0.08, 0.27)^***^
High dairy fat (*n* = 435)	0.02 (−0.07, 0.12)	−0.01 (− 0.11, 0.08)	0.15 (0.06, 0.24)^**^	0.09 (− 0.00, 0.18)

^*^p < 0.05; ^**^p < 0.01; ^***^p < 0.001

^a^Spearman’s correlation of energy-adjusted dietary intakes (g/d) with circulating 15:0 or 17:0 (% of total fatty acids), partially adjusted for age, sex, total energy intake;

^b^“Low total dairy” referring to subsample of patients with energy-adjusted total dairy intake below median intake of total sample and “high total dairy” to patients with energy-adjusted dairy intake at median intake or higher. Median energy-adjusted total dairy intake = 301 g/d

^c^“Low dairy fat” referring to subsample of patients with energy-adjusted dairy intake below median intake of total sample and “high dairy fat” to patients with energy-adjusted dairy intake at median intake or higher. Median energy-adjusted dairy fat intake = 12.0 g/d

## Discussion

In the present analyses of 869 post-MI patients of the Alpha Omega Cohort, total dairy and dairy fat intake were modestly correlated with circulating 15:0, but total fiber intake was not. However, both dairy and fiber intake were correlated with circulating 17:0. Correlations between dairy intake and circulating 15:0 and 17:0 were stronger when fiber intake was low. Likewise, correlations between fiber intake and circulating 15:0 and 17:0 were stronger when dairy intake was low. Circulating 17:0 was highest in patients with both high dairy and high fiber intake, while circulating 15:0 was highest in patients with high dairy intake, regardless of their fiber intake.

The strengths of the correlations of total dairy and dairy fat intake with circulating OCFA were comparable to those reported in a recent meta-analysis of 18 observational studies, mainly in general populations [1]. This meta-analysis reported weaker correlations with total dairy and dairy fat intake for 17:0 as compared to 15:0, which is confirmed by our data. Only few studies have reported correlations between circulating OCFA and intakes of both dairy and fiber-rich foods. Consistent with our findings, a cross-sectional study of 301 healthy Swedish men aged 60–64 years showed significant correlations of 17:0 in PL with intake of dairy fat (*r* = 0.23) and total fiber (*r* = 0.27) [25]. However, in contrast to our observations, PL 15:0 in these Swedish men was also significantly correlated with fiber intake (*r* = 0.20). In an EPIC-InterAct subcohort of almost 16,000 European adults, intakes of both dairy and fruits and vegetables were correlated with the sum of PL 15:0 and 17:0 (*r* = 0.1–0.2) [7]. As the proportion of 17:0 in PL tends to be 2–3 times higher than that of 15:0, the observed correlation with fruits and vegetables (key sources of fiber) were likely driven by 17:0, which would be in line with our findings. A cross-sectional analysis in 423 Finnish children aged 6–8 years old [26] found weak but significant correlations of 0.1 for 17:0 in PL and intakes of various high-fat dairy products such as milk (≥1% fat) and cheese (> 17% fat) as well as for 17:0 in PL and intake of grain products high in fiber (≥5% fiber). No association was found between 15:0 in PL and intake of high-fiber grain products in these Finnish children, which is also in agreement with our present study.

A randomized controlled trial that studied the effects of fiber intake on circulating PL 15:0 and 17:0 in 16 healthy participants found an increase of 17% in circulating 15:0 and an increase of 11% in circulating 17:0 after 1 week daily supplementation of 30 g inulin (a fermentable fiber). Although the average increase was larger for 15:0, it was more consistent for 17:0, with increases in PL 17:0 observed in almost all participants after inulin supplementation [13]. Based on these findings, the authors suggested that fermentation of fiber by gut microbiota could increase formation of propionate, a short chain FA, which would lead to hepatic synthesis of longer chain OCFA, in particular 17:0. The authors hypothesized that circulating OCFA could be a biomarker of fiber intake [13]. Our exploratory analyses showed that associations of dairy intake with circulating OCFA, particularly 17:0 in PL, were weaker in patients with a relatively high fiber intake. These findings suggest that circulating PL 17:0 may not adequately reflect dairy intake in populations with higher fiber intake. We also observed weaker correlations between circulating OCFA and fiber in patients with a relatively high dairy intake, which suggests that circulating OCFA may reflect fiber intake better when dairy intake in the population is low. It is noteworthy that in our exploratory analyses, circulating 17:0 was highest with high intakes of both dairy and fiber, and similar for high fiber or high dairy separately. Together, these findings imply that both dairy and fiber affect circulating proportions of OCFA, especially 17:0. This may also explain, for example, the lower correlation of 17:0 with dairy in high fiber subgroup. Higher fiber intake may contribute to higher circulating 17:0 from fiber, which could weaken the correlation of 17:0 with dairy intake.

Our findings may have implications for biomarker studies of nutrition and cardiometabolic diseases. Consistent inverse associations have been reported between circulating proportions of OCFA and cardiovascular-related outcomes [2, 11, 27], especially for 17:0, which would amount to an estimated 35% lower risk of type 2 diabetes according to a pooled analysis of 16 prospective cohort studies [2]. Observed risk reductions with higher levels of circulating OCFA have mainly been attributed to dairy intake [2, 7]. Our findings suggest that these associations, especially for 17:0 in PL, may be partly due to intake of dietary fiber.

We did not observe significant correlations between meat or ruminant meat intake and circulating OCFA, while fish intake was only weakly correlated with 15:0. This is not in line with an analysis from the EPIC-Oxford study, which reported a correlation of 0.4 between intake of ruminant meat and concentrations of 15:0 in PL [14]. This is also not in line with an analysis from the EPIC study in 16 European regions, which reported ecological correlations with 17:0 in PL of 0.4 for red meat and 0.7 for fish intake [28]. However, the latter analysis used mean values of intake and circulating OCFA in 16 European regions instead of individual data [28], and such aggregate data analysis is limited in adjustment for potential confounders. In our Alpha-Omega cohort, the intake of ruminant meat (18 g/d) was considerably lower than in the EPIC-Oxford cohort (30 g/d), which may be an alternative explanation why we did not observe an association with circulating OCFA. Furthermore, dairy intake in our cohort was relatively high, which reduces the relative contribution of ruminant meat to circulating OCFA. However, we did find a weak positive correlation between 15:0 in CE and fish, despite that median fish intake was low (14 g/d). Dietary fiber and fish were associated with higher circulating odd-chain fatty acids in our Dutch cohort, despite a relatively low fiber and fish intake. These findings need to be confirmed by other studies, preferably randomized controlled trials in populations with higher fiber and fish intake.

The present analysis has several strengths. We collected information about dairy intake using an FFQ that had been specifically designed for estimating fatty acid intakes [17, 18]. Fatty acids intake estimated by the FFQ were highly correlated to estimates by dietary history (Pearson’s correlation of ~ 0.8 for saturated and total fatty acids in g/d). We analyzed circulating OCFA both in PL and CE, which are two commonly used plasma fractions in epidemiological studies. Stability of FA composition over 6–9 years of storage in − 80 °C was confirmed and inter- and intra-assay variation for measurement of various fatty acids was < 8% in CE and < 5% in PL [23]. We examined circulating OCFA in relation to dairy intake in subgroups with high and low fiber intake. To the best of our knowledge, this approach has not been explored in other studies of circulating OCFA as biomarkers of dairy intake.

A limitation of our current study is that we could not accurately estimate the intake of specific types of dietary fiber, because the Dutch food composition table (NEVO, [19]) mainly reports on total fiber content of foods. Fiber intake in our cohort (~ 22 g/d) was in line with a Dutch survey in 2010–2012 among 739 individuals aged 70 years or older, showing mean fiber intakes of 22 g/d in men and 19 g/d in women [29]. These fiber intakes are below the recommended amount of 25 g/d for the Netherlands [30]. Possibly, if fiber intakes had been higher in our cohort, stronger associations with circulating odd-chain fatty acids could have been observed. Despite this limitation, we still observed correlations between circulating 17:0 and fiber, consistent with other studies. Another limitation is the cross-sectional nature of the study, meaning that observed associations cannot be interpreted as causal relationships. Higher fiber intake may be a proxy of better adherence to healthy diet and the possibility of residual confounding from other aspects of healthy diet cannot be fully excluded. Our cohort consisted of post-MI patients and 21% of our cohort had diabetes. The circulating FA composition may be affected by existing chronic conditions, such as insulin resistance, via changes in FA metabolism [31]. Nevertheless, means and ranges of circulating proportion of OCFA in PL and CE in these patients were similar to other studies in generally healthy populations [1]. Analyses on the differences in means of circulating 15:0 and 17:0 between combined groups of dairy and fiber intake were not prespecified and should therefore be considered exploratory.

## Conclusion

In this population of post-MI patients with a relatively high dairy consumption, circulating proportions of 15:0 were associated with dairy intake, but not fiber intake, whereas circulating 17:0 were associated with both dairy and fiber intake. The utility of 17:0 as a biomarker of dairy intake may depend on the amount of fiber consumed, and likewise, its utility as a marker of fiber intake may depend on the amount of dairy consumed. The data suggest that cardiometabolic health benefits previously attributed to biomarkers of dairy intake, especially 17:0, may partly be attributable to dietary fiber intake.

## Supplementary information

**Additional file 1: Table S1.** Individual dairy products included and grouping into high fat or low fat dairy for the present analysis of Alpha Omega Cohort. **Table S2.** Food sources of dietary fiber for the present analysis of Alpha Omega Cohort. **Table S3.** Spearman correlation coefficients for relation between intake and circulating odd-chain fatty acids additionally adjusted for medications and presence of type 2 diabetes. **Table S4.** Spearman correlation coefficients for relation between intake and circulating odd-chain fatty acids including only patients without type 2 diabetes (*n* = 683). **Figure S1.** Flow diagram for population for analysis. **Figure S2.** Associations of circulating 15:0 (A, B) and 17:0 (C,D) with dairy intake (energy-adjusted grams/day) evaluated by restricted cubic splines. **Figure S3.** Associations of circulating 15:0 (A, B) and 17:0 (C, D) with dairy fat intake (energy adjusted grams/day) evaluated by restricted cubic splines. **Figure S4.** Associations of circulating 15:0 (A, B) and 17:0 (C, D) with fiber intake (energy-adjusted grams/day) evaluated by restricted cubic splines.

## Data Availability

Datasets generated and/or analysed during this study are available in the DANS repository through the following link: 10.17026/dans-2a3-fg5q. The data catalogue of Alpha Omega Cohort is publicly accessible on www.alphaomegacohort.org.

## Abbreviations

CE: Cholesteryl esters
EPIC: European Prospective Investigation into Cancer and Nutrition
FA: Fatty acids
FFQ: Food frequency questionnaire
MI: Myocardial infarction
OCFA: Odd-chain fatty acids
PL: Phospholipids
