# Supplementary material for: Associations of dairy and fiber intake with circulating odd-chain fatty acids in post-myocardial infarction patients
Source: Nutr Metab (Lond). 2019 Nov 13;16:78. doi: 10.1186/s12986-019-0407-y (PMC6854617; doi:10.1186/s12986-019-0407-y)
Supplement: Supplementary file 1 — Additional file 1: Table S1. Individual dairy products included and grouping into high fat or low fat dairy for the present analysis of Alpha Omega Cohort. Table S2. Food sources of dietary fiber for the present analysis of Alpha Omega Cohort. Table S3. Spearman correlation coefficients for relation between intake and circulating odd-chain fatty acids additionally adjusted for medications and presence of type 2 diabetes. Table S4. Spearman correlation coefficients for relation between intake and circulating odd-chain fatty acids including only patients without type 2 diabetes (n = 683). Figure S1. Flow diagram for population for analysis. Figure S2. Associations of circulating 15:0 (A, B) and 17:0 (C,D) with dairy intake (energy-adjusted grams/day) evaluated by restricted cubic splines. Figure S3. Associations of circulating 15:0 (A, B) and 17:0 (C, D) with dairy fat intake (energy adjusted grams/day) evaluated by restricted cubic splines. Figure S4. Associations of circulating 15:0 (A, B) and 17:0 (C, D) with fiber intake (energy-adjusted grams/day) evaluated by restricted cubic splines. [file 12986_2019_407_MOESM1_ESM.docx]

Article: Associations of dairy and fiber intake with circulating odd-chain fatty acids in post-myocardial infarction patients

Authors : Pertiwi, Küpers, Wanders, de Goede, Zock, Geleijnse

Contents:

| Table S1 | Individual dairy products included and grouping into high fat or low fat dairy for the present analysis of Alpha Omega Cohort |
| --- | --- |
| Table S2 | Food sources of dietary fiber for the present analysis of Alpha Omega Cohort |
| Table S3 | Spearman correlation coefficients for relation between intake and circulating odd-chain fatty acids with additional adjustments for medications and presence of type 2 diabetes |
| Table S4 | Spearman correlation coefficients for relation between intake and circulating odd-chain fatty acids including only patients without type 2 diabetes (n=683) |
| Figure S1 | Flow diagram for population for analysis |
| Figure S2 | Associations of circulating 15:0 (A, B) and 17:0 (C, D) with dairy intake (energy-adjusted grams/day) evaluated by restricted cubic splines |
| Figure S3 | Associations of circulating 15:0 (A, B) and 17:0 (C, D) with dairy fat intake (energy-adjusted grams/day) evaluated by restricted cubic splines |
| Figure S4 | Associations of circulating 15:0 (A, B) and 17:0 (C, D) with fiber intake (energy-adjusted grams/day) evaluated by restricted cubic splines |

Table S1 Individual dairy products included and grouping into high fat or low fat dairy for the present analysis of Alpha Omega Cohort

| **Dairy products** | **Specific dairy food products** | **High fat or low fat dairy** |
| --- | --- | --- |
| Milk | Full fat milk | High fat dairy |
|  | Full fat chocolate milk | High fat dairy |
|  | Semi-skimmed and skimmed milk | Low fat dairy |
|  | Semi-skimmed and skimmed chocolate milk | Low fat dairy |
|  | Buttermilk | Low fat dairy |
|  |  |  |
| Yogurt | Full fat yogurt | High fat dairy |
|  | Semi-skimmed and skimmed yogurt | Low fat dairy |
|  | Yogurt drink | Low fat dairy |
|  |  |  |
| Dairy desserts | Full fat quark | High fat dairy |
|  | Semi-skimmed and skimmed quark | Low fat dairy |
|  | Full fat custard | High fat dairy |
|  | Semi-skimmed and skimmed custard | Low fat dairy |
|  | Other desserts with cream | High fat dairy |
|  | Full fat pudding | High fat dairy |
|  | Low fat pudding | Low fat dairy |
|  | Dessert porridge | Low fat dairy |
|  |  |  |
| Cream | Whipped cream | High fat dairy |
|  | Cream with warm meals (crème fraiche, sour cream) | High fat dairy |
|  |  |  |
| Cheese | All types | High fat dairy |
|  |  |  |
| Milk for coffee and creamers | Full fat milk added to coffee | High fat dairy |
|  | Semi-skimmed milk added to coffee | Low fat dairy |
|  | Full fat powdered milk for coffee | High fat dairy |
|  | Skimmed powdered milk for coffee | Low fat dairy |
|  | Full fat dairy based coffee creamers | High fat dairy |
|  | Skimmed dairy based coffee creamers | High fat dairy |
|  |  |  |
| Butter | Salted | High fat dairy |
|  | Unsalted | High fat dairy |
|  |  |  |
| Ice-cream |  | High fat dairy |

Table S2 Food sources of dietary fiber for the present analysis of Alpha Omega Cohort

| Fiber types | Food sources included | Mean ± SD | Median (IQR) |
| --- | --- | --- | --- |
| Grain fiber | Bread (whole wheat, white bread, multigrain, rye), buns, toast | 9.3 ± 3.6 | 9.0 (6.5-11.7) |
|  | Granola, cornflakes, fiber-rich breakfast products |  |  |
|  | Rice, noodles, pasta |  |  |
|  | Biscuits, cookies, pancakes, savory pie |  |  |
|  |  |  |  |
| Fruits fiber | Citrus fruits, apples, pears, bananas, strawberries, blueberries, cherries, grapes, peaches, nectarines, plums, apricot, kiwi, grapefruit, dried fruit, other fruit | 3.9 ± 3.6 | 2.7 (1.3-5.8) |
|  | Apple sauce |  |  |
|  | Orange juice, apple juice, grapefruit juice, other juices |  |  |
|  |  |  |  |
| Vegetable fiber | Endives, spinach, purslane, sprouts, cauliflower, broccoli, other cabbage, carrots, leek, chicory, kohlrabi, beets, mushrooms, bell pepper, onion, tomatoes, lettuce, crudités, other vegetables | 2.0 ± 1.1 | 1.8 (1.3-2.5) |
|  | Tomato juice, vegetable juice |  |  |
|  | Onion soup, tomato soup, other soup with vegetables |  |  |
|  |  |  |  |
| Potato fiber | Boiled potatoes, mashed potatoes, baked potatoes, hotchpot, potato salad, other potatoes, fries, potato chips | 3.7 ± 1.8 | 3.6 (2.4-4.6) |
|  |  |  |  |
| Beans, legumes, nuts fiber | Peas, broad beans, green beans, legumes, legume soup, various nuts | 1.6 ± 1.1 | 1.0 (0.5-1.6) |
|  |  |  |  |
| Other source (unknown) |  | 1.0 ± 0.8 | 0.8 (0.5-1.3) |

Table S3 Spearman correlation coefficients for relation between intake and circulating odd-chain fatty acids additionally adjusted for medications and presence of type 2 diabetes

|  | Fractions | Pentadecanoic acid (15:0) | Heptadecanoic acid (17:0) |
| --- | --- | --- | --- |
| *Dairy* |  |  |  |
| Total dairy | Phospholipids | 0.19 (0.13, 0.25)^***^ | 0.15 (0.09, 0.22)^***^ |
|  | Cholesteryl esters | 0.24 (0.17, 0.30)^***^ | 0.13 (0.07, 0.20)^***^ |
| Dairy fat | Phospholipids | 0.20 (0.13, 0.26)^***^ | 0.12 (0.05, 0.18)^***^ |
|  | Cholesteryl esters | 0.26 (0.20, 0.32)^***^ | 0.11 (0.05, 0.18)^***^ |
|  |  |  |  |
| Total fiber | Phospholipids | 0.07 (-0.00, 0.13) | 0.19 (0.12, 0.25)^***^ |
|  | Cholesteryl esters | 0.04 (-0.03, 0.10) | 0.11 (0.04, 0.17)^**^ |
| *Other foods* |  |  |  |
| Total meat | Phospholipids | -0.01 (-0.08, 0.05) | 0.01 (-0.05, 0.08) |
|  | Cholesteryl esters | -0.04 (-0.10, 0.03) | 0.02 (-0.05, 0.09) |
| Ruminant meat | Phospholipids | 0.06 (-0.01, 0.13) | 0.05 (-0.01, 0.12) |
|  | Cholesteryl esters | 0.05 (-0.01, 0.12) | 0.05 (-0.02, 0.11) |
| Total fish | Phospholipids | 0.02 (-0.05, 0.08) | 0.06 (-0.00, 0.13) |
|  | Cholesteryl esters | 0.09 (0.02, 0.16)^**^ | 0.02 (-0.05, 0.87) |

Partial *r_s_* are Spearman’s correlation coefficients between dietary intakes and individual circulating odd-chain fatty acids adjusted for age, sex, total energy intake, anti-hypertensive medication use, statins use and presence of type 2 diabetes;

^*^*p*<0.05; ^**^*p*<0.01; ^***^*p*<0.001.

Table S4 Spearman correlation coefficients for relation between intake and circulating odd-chain fatty acids including only patients without type 2 diabetes (n=683)

|  | Fractions | Pentadecanoic acid (15:0) | Heptadecanoic acid (17:0) |
| --- | --- | --- | --- |
| *Dairy* |  |  |  |
| Total dairy | Phospholipids | 0.17 (0.09, 0.24)^***^ | 0.14 (0.06, 0.21)^***^ |
|  | Cholesteryl esters | 0.23 (0.15, 0.30)^***^ | 0.17 (0.10, 0.24)^***^ |
| Dairy fat | Phospholipids | 0.20 (0.12, 0.27)^***^ | 0.10 (0.03, 0.18)^**^ |
|  | Cholesteryl esters | 0.27 (0.20, 0.34)^***^ | 0.15 (0.07, 0.22)^***^ |
|  |  |  |  |
| Total fiber | Phospholipids | 0.03 (-0.04, 0.11) | 0.18 (0.11, 0.25)^***^ |
|  | Cholesteryl esters | 0.02 (-0.06, 0.09) | 0.09 (0.01, 0.16)^*^ |
| *Other foods* |  |  |  |
| Total meat | Phospholipids | 0.02 (-0.06, 0.09) | 0.07 (-0.01, 0.14) |
|  | Cholesteryl esters | -0.01 (-0.09, 0.06) | 0.03 (-0.04, 0.11) |
| Ruminant meat | Phospholipids | 0.06 (-0.01, 0.14) | 0.10 (0.02, 0.17)^*^ |
|  | Cholesteryl esters | 0.05 (-0.03, 0.12) | 0.03 (-0.04, 0.11) |
| Total fish | Phospholipids | 0.01 (-0.09, 0.06) | 0.05 (-0.02, 0.13) |
|  | Cholesteryl esters | 0.07 (-0.01, 0.14) | 0.01 (-0.06, 0.09) |

Partial *r_s_* are Spearman’s correlation coefficients between dietary intakes and individual circulating odd-chain fatty acids adjusted for age, sex and total energy intake;

^*^*p*<0.05; ^**^*p*<0.01; ^***^*p*<0.001.

*n* = 4,837

*n* = 4,365

Implausible energy intake, *n* = 19

Missing dietary data, *n* = 453

Complete dietary data

*n* = 4,384

**Subsample with phospholipids and cholesteryl esters measurement**

*n* = 869

Plasma phospholipids not available, *n* = 3,476

Plasma phospholipids unknown>5%, *n* = 20

Plasma cholesteryl esters unknown>5%, *n* = 0

Figure S1 Flow diagram for population for analysis


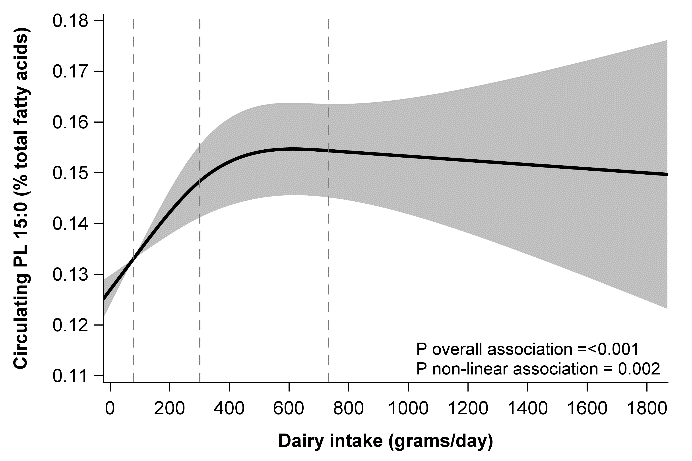


**B**

**A**


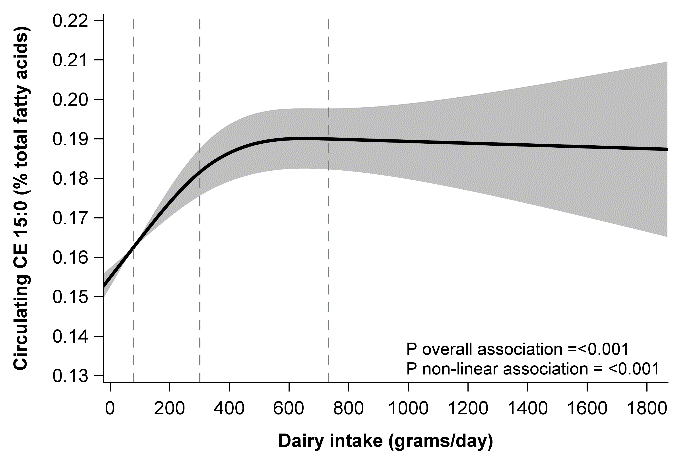


**C**

**D**


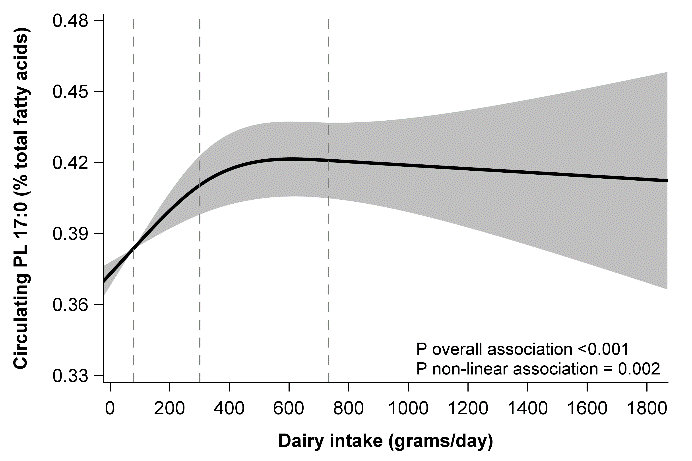

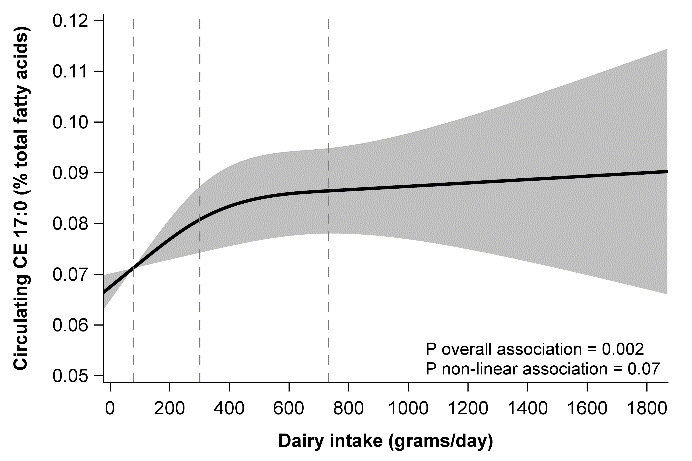


Figure S2 Associations of circulating 15:0 (A, B) and 17:0 (C,D) with dairy intake (energy-adjusted grams/day) evaluated by restricted cubic splines. Solid lines and grey areas are central estimates of circulating 15:0 or 17:0 and 95% confidence interval with adjustment for age, sex, and total energy intake. Knots were located at 5^th^, 50^th^ and 95^th^ percentile, represented by dashed vertical lines; Reference value was set at 5^th^ percentile of dairy intake which was 77 g/day;

CE, cholesteryl esters; PL, phospholipids.


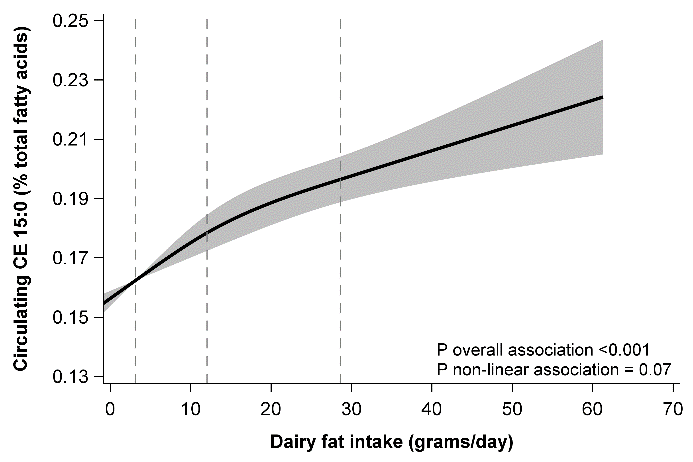

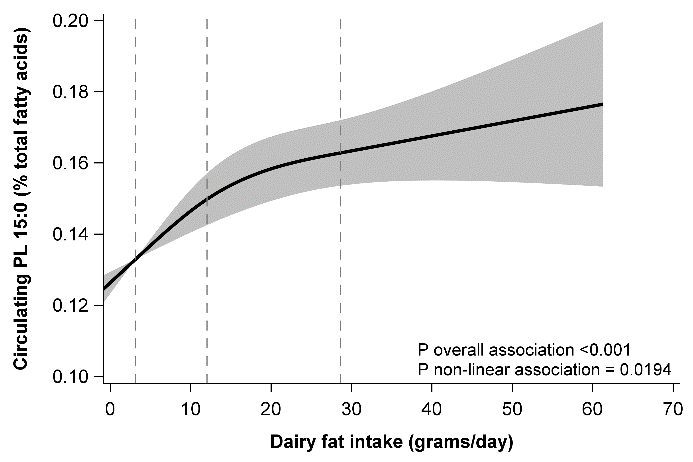


**B**

**A**

**C**

**D**

**
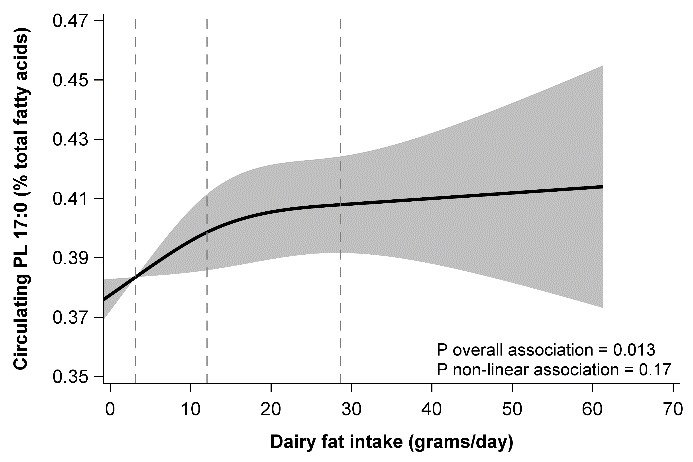

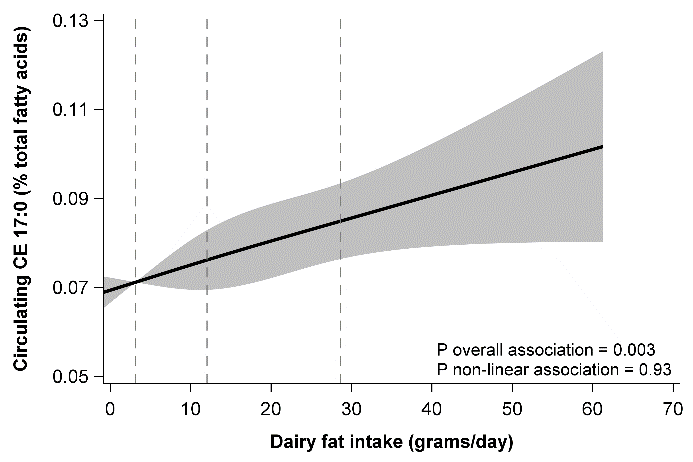
**

Figure S3 Associations of circulating 15:0 (A, B) and 17:0 (C, D) with dairy fat intake (energy adjusted grams/day) evaluated by restricted cubic splines. Solid lines and grey areas are central estimates of circulating 15:0 or 17:0 and 95% confidence interval with adjustment for age, sex, and total energy intake. Knots were located at 5^th^, 50^th^ and 95^th^ percentile, represented by dashed vertical lines. Reference value was set at 5^th^ percentile of dairy fat intake which was 3.2 g/day;

CE, cholesteryl esters; PL, phospholipids.


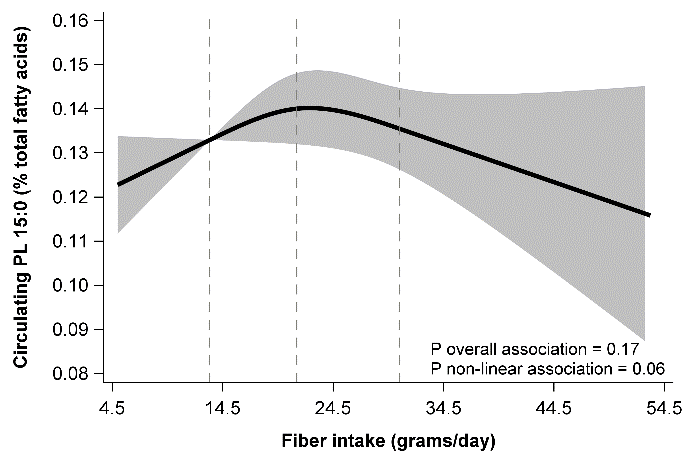


**B**

**A**


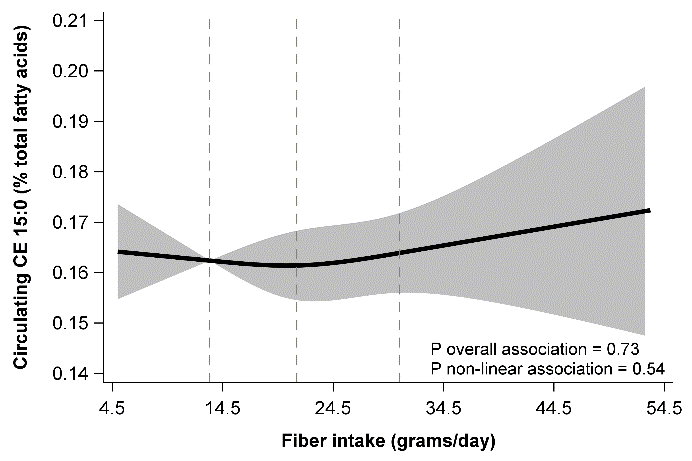


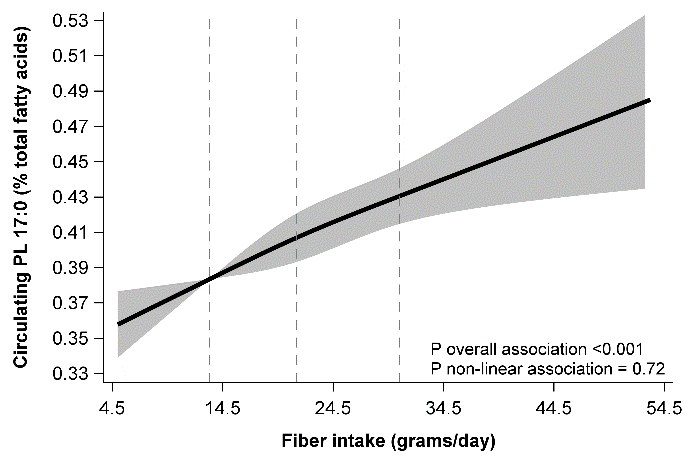


**D**

**C**


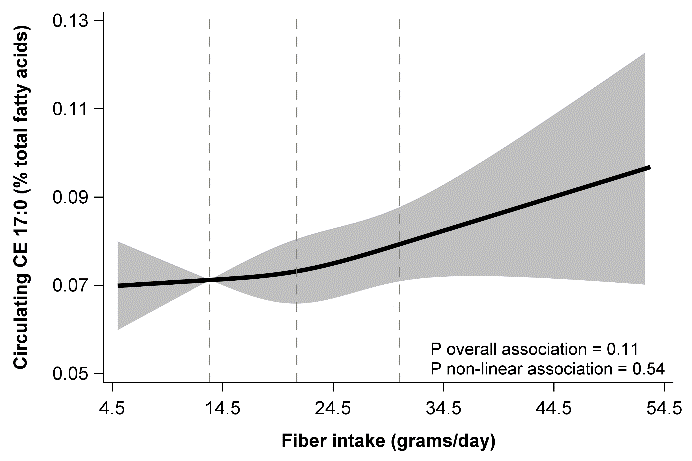


Figure S4 Associations of circulating 15:0 (A, B) and 17:0 (C, D) with fiber intake (energy-adjusted grams/day) evaluated by restricted cubic splines. Solid lines and grey areas are central estimates of circulating 15:0 or 17:0 and 95% confidence interval with adjustment for age, sex, and total energy intake. Knots were located at 5^th^, 50^th^ and 95^th^ percentile, represented by dashed vertical lines; Reference value was set at 5^th^ percentile of fiber intake which was 13.3 g/day;

CE, cholesteryl esters; PL, phospholipids.
